# Development of a community-based, one-stop service centre for children with developmental disorders: changing the narrative of developmental disorders in sub-Saharan Africa

**DOI:** 10.11604/pamj.2020.36.164.23402

**Published:** 2020-07-08

**Authors:** Yetunde Celia Adeniyi, Ade Fatai Adeniyi

**Affiliations:** 1 Department of psychiatry, College of Medicine, University of Ibadan and University College Hospital, Ibadan, Nigeria,; 2 Centre for Early, Development, Learning and Care, Ibadan, Nigeria,; 3 Department of Physiotherapy, College of Medicine, University of Ibadan and University College Hospital, Ibadan, Nigeria

**Keywords:** Developmental disorders, service development, children, sub-Saharan Africa, autism

## Abstract

Sub-Saharan Africa is home to about 15 million children with varying developmental disorders. Services for children with developmental disorders are scarce in Africa. The few available services are limited to the capital cities and are in the private sector, with the cost beyond the reach of most families. In 2016, the Centre for Early Development, Learning and Care was established in Ibadan, South-west, Nigeria. The centre provide services for children with developmental disorders is a one-stop, multidisciplinary team approach. Children are assessed on both structured and unstructured interviews. A total of 584 children were assessed between December 2016 and December 2019. One third (32.4%) of the children assessed within this period met diagnostic criteria for ASD, 29.1% had intellectual disability and 27.6% had cerebral palsy. The remaining clients (10.9%) had other disorders including ADHD, Down’s syndrome, hearing impairment and visual impairment. Parents tend to downplay the severity of their children’s disability. There are many challenges that are associated with the establishment of a service centre in a low resource setting. These and other experiences are discussed.

## Introduction

The Sustainable Development Goals (SDGs) call for an end to preventable deaths of new-borns and children by 2030, with all countries aiming to reduce neonatal mortality to at least as low as 12 per 1,000 live births, and under-5 mortality to at least as low as 25 per 1000 live births [1]. This is a follow up to the Millennium Development Goal 4 (MDG 4), Reduce child mortality, that called for the reduction of the under-5 mortality rate by two-thirds between 1990 and 2015 [2]. The combining efforts of these two programs have led to significant reduction in child mortality rate across the globe. According to the United Nations Children's Fund (UNICEF), 1 in 26 children died before reaching age 5 in 2017, compared to 1 in 11 in 1990 [3]. The drop in the rates is still significantly low in sub-Saharan Africa where about 2.7 million children died in 2017. The drop in death rate has resulted in more children surviving with some of them presenting with Neurodevelopmental disorders (NDDs). Health systems in sub-Saharan Africa (SSA), (mainly geared towards the control of infectious diseases), are less prepared to face the emerging challenges resulting from NDDs [4]. As a result, NDDs are often ignored or under-diagnosed with a high emotional and financial burden to the child and affected family.

Early childhood, commonly defined as the first 5 years of life, is the fastest period of growth and the period in which the developing brain is most sensitive to stimulation and nurturing [5, 6]. Interventions are most effective during this period [5]. The most relevant and widely reported data to date suggest that roughly 250 million children are at risk of suboptimal development in low-and medium-income countries (LMICs) [7]. Olusanya *et al*. reported that the number of children younger than 5 years with epilepsy, intellectual disability, sensory impairments, autism spectrum disorder, and attention-deficit hyperactivity disorder in SSA rose from 8.6 million in 1990 to 14.7 million in 2016. The highest YLDs for all disabilities except for ASD were reported in sub-Saharan Africa [8]. Services for children with developmental disorders are scarce in Africa [9, 10]. The few available services are limited to the capital cities and are in the private sector, with the cost beyond the reach of most families [10].

**The situation and pathway to care for individuals with developmental disorders in Ibadan Nigeria before the centre:** Ibadan is the largest city in West Africa. It has a population of about 3,532,400 [11] and approximate land area of 250 km^2^. The first University in Nigeria is situated in the city with the teaching hospital arm known as the University College Hospital (UCH). Mental health services are provided by the university teaching hospital with child and adolescent mental health services, which became a stand-alone service about 10 years ago. There are five Child and Adolescent Psychiatrists in the city, about five people working as speech and language therapists (only one of this is in the teaching hospital, the remaining work in the community), three occupational therapists and no psychologist with child and adolescent mental health (CAMH) training. There is no documented community prevalence of developmental disorders in Nigeria. However, there are reports from studies done among high risk groups that showed that the prevalence could be as high as 40% among children in institutions and clinics [12]. Majority of the children with developmental disorders in sub-Saharan Africa are not diagnosed and do not receive care.

The pathway to care for most children in south-west, Nigeria is usually through faith-based organisations and mainstream schools, with a tiny fraction of them getting formal assessment at the UCH. The primary and secondary health care system is almost non-functional in terms of service provision for developmental disabilities. Families often recognise signs of developmental delays early but do not seek care due to unavailability of appropriate services. This often continues until the children start elementary school at about 6 years and are noticed by their teachers. Sometimes they are referred by teachers to health professionals. However, developmental disorders are often missed, even by health professionals who are not specialists. Many people with developmental disorders have co-morbid mental health problems like anxiety, depression and psychosis and therefore come into contact with mental health services. It is usually at this presentation that their developmental disorders are diagnosed for the first time. Unfortunately, diagnosis in most part of Africa does not necessarily translate to adequate treatment due to lack of appropriate care and therapy centres for developmental disorders.

## Project evaluation

**Starting the centre:** one of the authors, YCA, who is also the lead clinician, had an initial training as a psychiatrist (Fellow of West African College of Physicians) and later trained as a Child and Adolescent Psychiatrist through a 2-year sub-specialty program under the National Postgraduate Medical College of Nigeria after which she was awarded the Advanced Certificate in Child and Adolescent Psychiatry. She is one of the only two with this qualification in the entire country. During this sub-specialty training, she was working in the outpatient and inpatient services of the Department of Child and Adolescent Psychiatry, UCH. She was also volunteering in homes and institutions that take care of children with developmental disorders and other mental health issues across the city. Her Master of Science work was also among children with intellectual disability [13]. During the years of postgraduate trainings, the author observed obvious lack of appropriate care for children with NDDs.

The key step in establishing a child development centre is to gather a group of like-minded people who are passionate about the cause. In December 2016, YCA, a Child and Adolescent Psychiatrist, a physiotherapist (AFA), two Special Education teachers, a speech and language therapist, a secretary, two support staff and a guard began the centre. There was just enough fund to rent a space and purchase the initial items and equipment needed for the centre. The mission, vision and core values of the centre were established. Our vision is for every child with developmental disability to reach his/her full potential and live a meaningful life. It is our mission to bring out the best in every child living with developmental disabilities and support their families through evidence based and affordable programs. We set out to achieve this through our four-part mission: *clinical and biomedical services:* to provide a well-coordinated, multidisciplinary, and affordable care to persons with developmental disabilities and their families; *training:* to provide training for capacity building; *Research:* to conduct innovative and culturally-sensitive research and *mental health promotion and policy*: to partner with stakeholders to impact mental health promotion, policy and advocacy.

Some of our core values are: person and family-oriented services, cultural competence and diversity, community inclusion, early intervention, Evidence-based practice and collaboration, Building capacity through training and culture-sensitive research. As at March 2020, the team has enlarged and consisted of the following with their usual time allocation: clinical lead and director: consultant child and adolescent psychiatrist-4 hours daily; professor and head of physiotherapy-3 hours daily; physiotherapist-4 days; speech therapist-full time; speech therapist-part time; occupational therapist-full time; 8 special education tutors-full time; music and art tutor-full time; hostel supervisor-full time; administrator and head of operations-full time; 13 support staff-full time; 3 security guards-full time and volunteer-4 hours weekly.

As the centre has become more established, it has been a popular destination for individuals and professionals with interest in developmental disorders. The Centre has received visitors and undertaken trainee placements from both undergraduate and postgraduate students, and doctors from the United Kingdom, United States of America, Australia, Canada, South Africa and many African countries. Prior to commencement of operations at the centre, the accreditation team from the Ministry of Women Affair and Social Inclusion visited the centre. The centre was subsequently approved to commence full operation, with an approval letter from the commissioner in charge of the ministry.

**Physical space and equipment:** the plan was to provide core therapy for children with developmental disorders including boarding and respite care. For ease of coordination, we had to get an apartment that will accommodate both the therapy sessions and hostels. A storey building apartment was rented for a year at the start, and it´s been renewed yearly since 2016. We had to carry out lots of structural partitioning of the apartment to accommodate the different therapy units. Due to limited funds, we bought the most needed equipment at the beginning. These were sourced locally and we gradually update as the need arises.

**Referrals:** we accept referrals from many sources. But most of our referrals came from: the teaching hospitals and other government hospitals in town; from NGOs, faith-based organisations and other outfits that work with children within the community; mainstream schools; snowballing of referrals based on testimonials from parents who already have children in the centre. This constitutes over 50% of referrals to the centre and self-referrals through social media, sign posts and various awareness programs (Table 1).

**Table 1:** steps in assessment and placement

Step	Domain	Components
1	An initial comprehensive interview and mental state assessment	Biodata History of disability Developmental history Family history Past medical history; surgery, medication use History of past intervention, therapy, education. Diagnosis with the DSM-V
2	Structured interview: use of specific tools	Bayley screening Conners Parents Rating Scale MCHAT Strengths and Difficulties questionnaire Wechsler Intelligence Scale for Children Patient Health Questionnaire-9
3.	Assessments for physical rehabilitation	Speech assessment Adaptive functions Physical therapy
4	Meeting of professionals	Agree on the diagnosis Identify areas of deficit
5	Meeting with caregivers and Child	Development of Individualised Care plan Placement
6	Placement, development of IEP and curriculum	Early Intervention, Special Education/Vocational skills, physiotherapy and stimulation groups

**Assessment tools:** the following assessment tools are used at presentation: Bayley´s scales of infant and toddler-third edition: It is a comprehensive instrument to examine the different aspects of development in children ages 1-42 months. It involves interaction between the child and examiner and observations in a series of tasks. It takes between 30-90 minutes to administer [14]; Wechsler Intelligence Scale for Children-Fourth Edition (WISC-IV): For the assessment of intelligence quotient of the clients [15]. Modified Checklist for Autism in Toddlers (MCHAT): Evaluates risk for ASD in children ages 16-30 months [16]; Conners´ Parent Rating Scale: It is a measure of behavioural problems associated with ADHD [17]; Patient Health Questionnaire 9 (PHQ-9): Short Depression Rating Scale [18] and Strengths and Difficulties Questionnaire (SDQ) [19]:

**Initial assessment:** this usually occurs within 48 hours of contact with the centre and is usually conducted by the lead clinician. It lasts between 60 minutes and 90 minutes. The assessment follows a semi-structured manner using the Diagnostic and Statistical Manual-Fifth Edition (DSM-V). This is usually done during the first appointment. The MCHAT, Conners Rating Scale, SDQ and PHQ are administered at this visit. The speech and language pathologist and a special education teacher will also carry out appropriate assessments. The physiotherapist assesses the clients with physical disabilities.

**Developmental assessment:** the primary caregiver, usually the mother is interviewed on pregnancy, delivery, neonatal and childhood history. Other history includes family, past medical and previous intervention, or care. The Bayley´s screening tool and WISC-IV are used at this stage. This usually lasts about 90 minutes on the average. We sometimes interview other family members like the grandparents and teachers, and this is usually done through phone calls if they did not accompany the parents. School report and medical reports are also reviewed where available. After the assessment, the team will meet to compare findings and take a decision about diagnosis, admission, and placement. Further interview is needed if the family is considering boarding facility. Reason for the choice of boarding, financial implications, and the need for continuous interaction between the centre and family while the child is in the hostel is discussed.

**Placement:** after the assessment, children are either placed in the day or boarding (residential) program based majorly on the family´s preferences. The day program involves that the children are brought to the centre every morning from Monday to Friday between 8am and 5pm. During this time, the children receive different interventions based on their needs.

**Figure 1:**
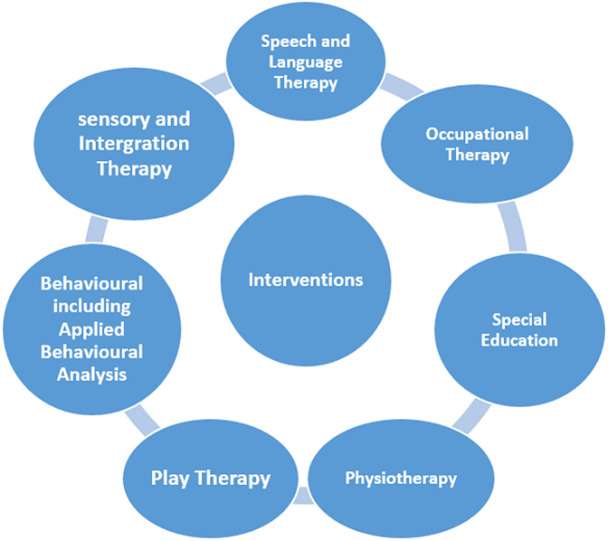
available interventions at the centre

**Available intervention and services at the centre:** we offer core services such as speech and language therapy, occupational therapy and physiotherapy. Others are shown in Figure 1.

**Some other services provided by the centre:** biomedical service: the centre provides basic medical services for the children at the centre, especially those in the boarding house. About 80% of the students have one or more comorbidities that require medications. The commonest comorbidity is epilepsy accounting for about 70% of the cases, and others include ADHD and sleep problems. Medications are administered and reviewed for students in the centre. Referrals are made to the teaching hospital for specialists care in some cases, for example, poorly controlled epilepsy is often referred for the neurologist´s review.

**Residential care:** the centre provides residential services to students especially children outside the city of Ibadan, where the centre is located. The boarders stay in the hostel for average of 12 weeks after which the centre goes on a few weeks break necessitating that the children go home to their families. They are fed and cared for at the hostel by trained caregivers with supervision from the therapists. The boarders also join the day program from 8am to receive therapy and return to the hostel in the evening. They also continue to receive interventions especially behavioural and self-help skills in the hostel.

**Education and training:** one of our mission is to provide structured training facilities for students and professionals working with children and adolescents. Students from Universities and tertiary institutions come for observership programs. In addition, family education and training aimed at improving understanding of relevant health issues to aid long-term compliance with treatment is organised periodically. The centre also provides continuous in-service training for staff of the centre.

**Research:** one of the objectives of the centre is to conduct research on developmental disorders with emphasis on developing countries. This started with cross sectional studies with a plan to subsequently recruit patients into clinical trials and facilitate follow up of patients in long-term trials.

## Discussion

**Figure 2:**
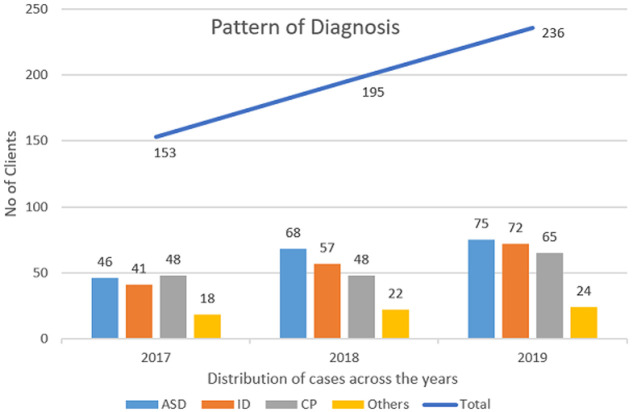
distribution of cases across the years

As at December, 2019, the centre has assessed 584 clients. One third (32.4%) of them met diagnostic criteria for ASD, 29.1% had intellectual disability and 27.6% had cerebral palsy. The remaining clients (10.9%) had other disorders including ADHD, Down´s syndrome, hearing impairment and visual impairment. See Figure 2 for the distribution of cases across the years. One major recurring issue that we have noticed is that parents tend to downplay the severity of disability of their children. This is often expressed by parents´ reluctance to allow their children interact with other children in the centre with the fear that the other children will negatively influence their children and by so doing worsen the condition of the child. Whereas, in most instances, based on expert analyses, their own children presented with severer cases. The team mostly resolve this by explaining the concept of individualised care and one-on-one approach to therapy.

**Figure 3:**
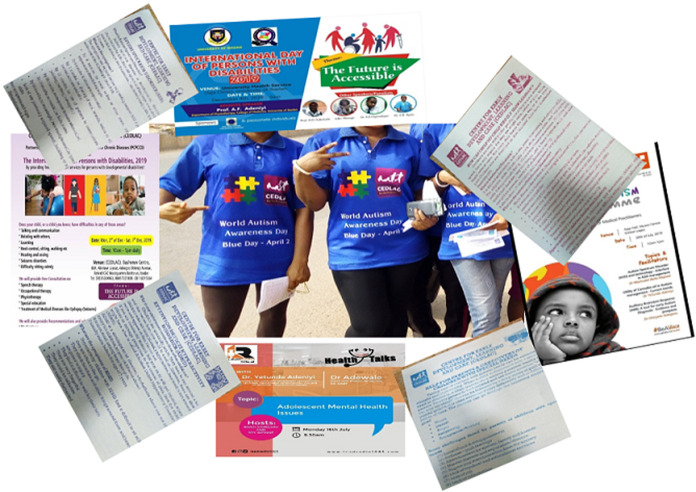
efforts on public awareness and advocacy

Students´ progress in the centre are monitored by the therapists in charge and therapists give weekly report of the students to the Head of Therapy. A 3-monthly comprehensive report is provided for parents, the report often include response to the different interventions, difficulties encountered and recommendations. The centre collaborates with mainstream schools in the city to allow for smooth transition of children from the centre to the mainstream education for inclusion. We provide a summary of activities and interventions the child had gone through, a summary of improvements and recommendations to support the child in the inclusive setting (Figure 3).

An important part of our objective is to provide education and awareness on developmental disorders, and drive advocacy. This objective is being achieved through several channels including: production and distribution of educational materials to members of the community in the form of leaflets written in clear and simple language. These leaflets provide information on common developmental disorders, symptoms, causes, and interventions. Since inception, the centre has distributed over 30,000 pamphlets;

**Television and radio:** there has been tremendous effort from the centre on public awareness through the local television and radio stations. Some of the slots were paid for by the centre, but most of the slots were by invitation from the television and radio stations. People in the community had opportunities to phone-in during some of the programs to ask questions and make necessary enquiry about developmental disorders. Some of these programs were in local dialects, providing opportunities for non-English speaking population;

**Social media:** our social media handles have served as sources of information to the general public on developmental disorders. This is especially more elaborate during the public awareness days that are relevant to developmental disorders like the Autism Awareness Day.

**Public talk in religious and social groups:** as a result of the important role the centre is playing in advocacy and awareness, we have received numerous invitations to speak at different social gatherings on issues relating to developmental disorders and other programs like community outreach to provide free developmental assessment and medical advice to children with developmental disorders and their families.

**Strengths of the centre:** the centre provides appropriate mental health support for both the child and family members through counselling, psychotherapy, assessment, and management of different mental illnesses for caregivers.

**One stop:** burden of health care in Nigeria and most African countries rest totally on the family. Families pay out of pocket for services. Services are scarce and poorly coordinated. The Centre provides well-coordinated services for children with special needs.

**Serves as a model centre:** the centre´s multidisciplinary assembly of staff, with multilevel professional training and experience in addition to a wide network of collaborations and partnerships and one-stop setting, makes it a model for others in the region.

**Challenges:** one of the recurrent challenges has to do with availability of qualified professionals. Professionals working in the field of disabilities are scarce in countries like Nigeria. The few ones are constantly looking for greener pasture, hence the personnel turnover rate is remarkably high. We have had to hire ad hoc staff to meet up with services because of sudden resignation or exit of therapists. The need for re-training and continuous medical education of staff. The centre is a private establishment with initial funding solely from the founders. The initial rent, furniture, therapy materials and initial salary came from the founders of the centre. Subsequent funding came from the fee paid by the parents. Students are billed based on their need for therapy, with quite a number of the families not able to pay for all the required therapy.

**Feedback and evaluation:** parents are encouraged to evaluate the different segments of our services through a feedback form. Evaluation follows both process-based and outcome-based. Some of the feedback from the parents are in Annex 1. We also ask our visitors to evaluate the setting and structures during their visits, and some of their comments are in Annex 2.

## Conclusion

A little over three years of commencement, the Centre has continued to grow and in the process, we have learnt a lot of lessons to help us improve our services. The number of families we serve, the number of regions we cover has progressively increased over the years. We have continued to evaluate our services and get feedback from users and partners. We are grateful to the community in which we operate as they have accepted and supported us in no small measure. We are indebted to the friends and partners of the centre for their advice, feedback, criticisms, and support to improve our services. With a clear vision, we are poised to continue to provide evidence-based, affordable, and culturally acceptable services to the children with developmental disabilities and their families.

## Annex

**Annex 1**: parents´ feedback

**Annex 2**: comments from visitors

## References

[1] United Nations Development Programme Sustainable Development Goals.

[2] United Nations Development Programme Millennium Development Goals.

[3] United nation Children Education Fund (UNICEF) Levels and Trend in Child Mortality.

[4] Baart J, Taaka F (2017). Barriers to healthcare services for people with disabilities in developing countries: A literature review. Disability, CBR & Inclusive Devpt.

[5] Pia R Britto, Stephen J Lye, Kerrie Proulx, Aisha K Yousafzai, Stephen G Matthews, Tyler Vaivada (2017). Nurturing care: promoting early childhood development. The Lancet.

[6] World Health Organization Early Child Development.

[7] Susan Walker P, Theodore Wachs D, Sally Grantham-McGregor, Maureen M Black, Charles A Nelson, Sandra L Huffman (2011). Inequality in early childhood: risk and protective factors for early child development. The lancet.

[8] Global Research on Developmental Disabilities Collaborators (2018). Developmental disabilities among children younger than 5 years in 195 countries and territories 1990-2016 a systematic analysis for the Global Burden of Disease Study 2016. The Lancet Global Health.

[9] Muideen Bakare O, Kerim Munir M, Mashudat Bello-Mojeed A (2014). Public health and research funding for childhood neurodevelopmental disorders in Sub-Saharan Africa: a time to balance priorities. Healthc Low Resour Settings.

[10] Tekola B, Baheretibeb Y, Roth I, Tilahun D, Fekadu A, Hanlon C (2016). Challenges and opportunities to improve autism services in low-income countries: lessons from a situational analysis in Ethiopia. Glob Ment Health (Camb).

[11] World Bank (2020). United Nations-Statistical data.

[12] Yewande Oshodi O, Andrew Olagunju T, Motunrayo Oyelohunnu A, Elizabeth Campbell A, Charles Umeh S, Olatunji Aina F (2017). Autism spectrum disorder in a community-based sample with neurodevelopmental problems in Lagos, Nigeria. J Public Health Afr.

[13] Yetunde Adeniyi C, Olayinka Omigbodun O (2016). Effect of a classroom-based intervention on the social skills of pupils with intellectual disability in Southwest Nigeria. Child Adolesc Psychiatry Ment Health.

[14] Bayley N (2006). Bayley scales of infant and toddler development: Bayley-III.

[15] Wechsler D (1949). Wechsler Intelligence Scale for Children, Fourth Edition (WISC-IV).

[16] Robins DL, Fein D, Barton M (1999). The Modified Checklist for Autism in Toddlers (M-CHAT).

[17] Conners CK, Sitarenios G, Parker JD, Epstein JN (1998). The revised Conners' Parent Rating Scale (CPRS-R): factor structure, reliability, and criterion validity. J Abnorm Child Psychol.

[18] Kroenke K, Spitzer RL, Williams JB (2001). The PHQ-9: validity of a brief depression severity measure”. J Gen Intern Med.

[19] Goodman R (1997). The Strengths and Difficulties Questionnaire: A Research Note. Journal of Child Psychology and Psychiatry.

